# Food Labeling: Analysis, Understanding, and Perception

**DOI:** 10.3390/nu13010268

**Published:** 2021-01-19

**Authors:** Daniela Martini, Davide Menozzi

**Affiliations:** 1 Department of Food, Environmental and Nutritional Sciences (DeFENS), Università degli Studi di Milano, 20133 Milan, Italy; 2 Department of Food and Drug, University of Parma, 43124 Parma, Italy

Food labels are the first informative tool found by the customers during shopping, and are informative in terms of ingredients, nutrient content, and the presence of allergens of the selected product. However, food labeling also represents a marketing tool and may influence perception of the food quality and, in turn, the dietary choice of consumers. For this reason, there is growing research in the food labeling field and in the evaluation of its effects on consumers, food operators, and the whole market [1,2]. This is supported by a wide range of manuscripts published in recent years, for instance, with the specific purpose to better investigate how specific information on the food packaging may influence food purchases and consumption and, in general, dietary behavior [3,4,5,6,7,8]

The Special Issue “*Food Labeling: Analysis, Understanding, and Perception*” was conceived with the intention to further explore current efforts in food labeling research and welcomed original studies, as well as reviews of the literature, focusing on: (i) the analysis of the nutrient profile of products with different characteristics reported on the food labels, i.e., nutrition and health claims (NHCs), organic, gluten-free (GF); (ii) the nutrient profile underlying front-of-pack (FOP) nutrition labels and their graphical design in different countries; (iii) the consumers’ perception, knowledge, and understanding of the information provided on food labeling; (iv) the impact of information on food labeling (e.g., FOP information, serving size) on consumers’ willingness to pay and food choice; (v) the attitudes, beliefs, and perceptions and behavioral and socioeconomic determinants regarding the use of food labels.

This Special Issue provides a series of 25 contributions, with 20 original papers, four narrative reviews, and one commentary. This last article is a consensus by eminent exponents of the International Carbohydrate Quality Consortium (ICQC) [9], which underlines the importance of dietary fiber, which is not always mandatory on food labeling (e.g., Reg. (EU) No 1169/2011) [10]. The authors supported the need for including fiber values in food labeling by distinguishing between intrinsic and added fiber, which may also help to achieve the recommended intake by consumers. The need to consider other information on food labels has also been discussed by Marinangeli and colleagues [11], who reviewed the regulatory frameworks and examples of associated non-mandatory food labeling claims currently employed to highlight healthy carbohydrate foods to consumers. Among the information, the authors considered NHCs related to dietary fiber, glycemic index, and glycemic response, and the presence of whole carbohydrate foods and ingredients that are intact or reconstituted (e.g., whole grains).

Some studies focused on the analysis of the nutritional quality of specific food groups and/or specific nutrients. Three studies were performed within the Food Labeling of Italian Products (FLIP) [12,13,14], a project aiming to evaluate the nutritional quality of packaged products currently sold in the online shops of several retailers in Italy [15,16,17]. Specifically, two studies focused on the analysis of the food labeling of breakfast cereals [12] and pasta [14]. The first study reported an elevated inter-product variability among breakfast cereals currently sold in Italy, with only limited differences when products with NHCs and GF declarations were compared with products not carrying this information [12]. Similarly, the study performed on pasta revealed that pasta types currently on the Italian market largely vary in terms of nutrition profile, with stuffed pasta characterized by a high salt content [14]. This last aspect supports the importance of providing nutrition facts of product to consumers to help them in making informed food choices. The last study performed within the FLIP project compared the nutritional quality of organic and conventional food products, highlighting that, with just a few exceptions, prepacked organic products are not of a superior nutritional quality than conventional ones, based on the mandatory information present on their packaging [13].

Yusta-Bojo et al. [18] focused on the sugar content in the most-consumed processed foods in Spain and compared the sugar values declared on the label (LVs) with laboratory analysis values (AVs). The study findings evidenced a high adequacy of LVs with the EU labeling tolerance requirements, with only cured ham presenting significant differences between the median AVs and LVs. Lastly, Azzopardi et al. investigated the energy density (ED) of food products targeted at children sold in Australia, finding a high proportion of products with a high ED (i.e., >950 kJ/100 g) among the 548 food items considered [19]. The same study observed that the health star rating (HSR) system, one of the FOP systems introduced in Australia in 2014, did not consistently discriminate between ED levels, particularly for high-ED foods. 

The HSR was also studied in another study focused on consumers’ perception [20] and performed with fifteen Australian grocery shoppers. Intriguingly, the findings from this study showed that the HSR was perceived as a simple, easy-to-understand, and useful tool, despite a certain grade of skepticism concerning its conception. The consumers’ perception and responses to FOP labels was also considered in another two papers published by Egnell et al. [21] and Talati et al. [22], showing results from the Netherlands and across another 12 countries, respectively, supporting that this represents a widely explored field of research. In detail, the first study [21] compared the perception and understanding of five FOP labels (HSR, Nutri-Score, multiple traffic lights (MTLs), reference intake, and warning symbols) among 1032 Dutch participants, finding a favorable perception, with Nutri-Score showing the highest performance in helping consumers to rank the products according to their nutritional quality. Conversely, in a similar study performed with over 12,000 participants across 12 countries, MTLs obtained the most favorable ratings, with mixed or neutral perceptions of the other FOP labels [22]. A third study by Breen et al. [23] compared NHCs, the HSR, and the price of snack foods sold in health food (HF) stores and aisles with the ones sold in regular areas (RAs) of supermarkets. The results showed that snack foods of HF stores displayed a significantly higher number of product claims compared to RA foods, together with a higher HSR and cost.

Botelho et al. [24] analyzed the FOP of food items shown in specific sections of the circulars of two Brazilian supermarket chains during a 10-week period, classifying them by their “unprocessed/minimally processed” versus “ultraprocessed” (UP) items and the presence and type of claims on the FOP. The NOVA systems represent another way of classifying foods that has receiving growing interest and which is based on the degree of food processing [25]. In this Special Issue, authors found that more than 50% of the items sold in the health and wellness section were UP and reported a high presence of reduced and increased nutrient content claims, suggesting that supermarkets’ circulars often promote the sale of UP foods.

Besides the study of consumers’ perception of FOP, it is worth investigating the predictors of consumer interest in FOP and back-of-pack labels. This was the object of a Polish study [26] which found that self-rated knowledge about nutrition healthiness is the only significant predictor in over 1000 Polish consumers, while neither demographic nor socioeconomic variables were significant predictors of interest towards food labels. Plasek and coworkers [27] focused on six categories of actors that seem to influence the perceived healthiness of foods: (i) the communication information (such as FOPs and NHCs), (ii) the product category, (iii) the shape and color of the product packaging, (iv) the ingredients of the product, (v) the organic origin of the product, and (vi) the sensory characteristics of food. Bryla [28] also found that FOP label reading is one of the predictors of the importance linked to salt content in over 1000 Polish consumers, in addition to other predictors such as the importance and attention to NHCs and the respondent’s age. 

Two studies applied hypothetical discrete choice experiments to analyzing consumers’ choices and willingness to pay (WTP) for, respectively, fish products [29] and pork sausages [30]. Menozzi and colleagues interviewed 2500 fish consumers in five European countries to assess the relative importance and WTP for different fish species and labeled attributes (i.e., sustainability label, NHCs, product presentation, production system, and price). The findings showed positive premiums for sustainability label, NHCs, and wild-caught alternatives, with high heterogeneity across countries and species [29]. Czine et al. [30] investigated whether product characteristics indicated on food labels of sausage made from traditional Hungarian mangalica pork might influence consumers’ choices. The authors found respondents’ preference for the label of origin indicating meat from registered animals, and purchasing from the farmers’ market is preferred over the butcher and hyper-/supermarket. 

Country-of-origin (COO) labeling effects were analyzed by Bimbo et al. [31]. The authors tested the price differential associated with the COO information for extra-virgin olive oil (EVOO) in Italy, employing a hedonic price model on the purchase of EVOO products collected from 982 consumers at the supermarket checkouts. Although the mandatory COO labeling regulation for EVOO can be an effective tool for consumers to identify the origin of the product and for producers to differentiate products, the results evidenced a significant share of consumers unable to correctly identify the origin of the EVOO purchased, mostly among consumers who reported having purchased Italian EVOO. 

Two experiments were conducted to analyze the effects of visual aids and color nutrition information (CNI) on sugar-sweetened beverages [32] and sweet food consumption [33]. Merillat et al. [32] assessed the effects of visual aids on judgments of sugar quantity in popular drinks and the choices of 261 individuals recruited in the USA. In the experimental condition, participants viewed beverages along with test tubes filled with the total amount of sugar in each drink and this led to a lower intention to consume any of the beverages, suggesting that this simple visual aid intervention affected judgments and choices towards curtailing sugar intake. Using an eye-tracking technique, Potthoff et al. [33] evaluated the effect of CNI based on a traffic light system adopted in Austria; participants in this study viewed images depicting sweets preceded by a colored circle informing about the sugar content of the food, with and without nutrition information. The results showed that the intervention had the opposite of the intended effect and the authors questioned whether CNI is helpful to influence initial cue reactivity toward sweet foods.

A quasi-experimental online trial on the choice of sugar foods was performed by Chen et al. [34] in Taiwan. The authors analyzed how mothers’ choices of low-sugar food were affected by theory-driven nutrition interventions, finding that, after the intervention, they exhibited enhanced sugar and nutrition label knowledge, perceived behavioral control, behavioral intentions, and behavior. 

Another experiment was conducted by Modlinska et al. [35] with 99 Polish individuals to assess the influence of food labeling (insect content) and appearance (traces of insect-like ingredients) on the participants’ perception. The results showed that products labeled as containing insects are consumed with reluctance and in lower quantities despite their appearance, regardless of the form in which the insects are served. The authors provided recommendations for labeling strategies to help to reduce the effect of disgust.

As already mentioned, food labeling does not include only nutritional information, and this is why a series of papers focused on other aspects is included. For instance, Ontiveros and colleagues [36] focused on allergens, by evaluating the characteristics of food allergen labeling and precautionary allergen labeling (PAL) in over 10,000 products sold in six Latin American countries. The authors found a high (>87.4%) compliance with local regulations, but countries without specific regulations for allergen labeling had two-fold more products containing allergens in their ingredients lists but no food allergen labeling, compared to countries with regulations. These results suggest that the lack of regulations for the characteristics of allergen labeling increases the risk of accidental exposure to allergens of interest.

Another interesting topic was reviewed by Van der Horst and coworkers [37], who investigated how healthy adults perceive and interpret serving size information on food packages and its influence on product perception and consumption. In their systematic review, the authors observed an overall poor conception of serving size, while the few included studies showed that labeled serving size affects portion size selection and consumption.

Finally, Rincón-Gallardo Patiño et al. [38] investigated restaurant menu labeling policies and their effects on menu reformulation. The authors found three voluntary and eight mandatory menu labeling policies primarily for energy disclosures, developed in upper-middle- and high-income countries, whereas none was found in low- or middle-income countries. The subsequent analysis conducted by the authors showed reductions in energy for newly introduced menu items only in the US. Implications for policy, practice, and research are also provided.

Overall, the studies included in the Special Issue provide new insights in this field of research, with relevant recommendations for policy makers, business operators, and researchers for developing more effective labeling strategies, allowing consumers to make informed dietary choices. At the same time, many authors reported the need for performing further investigations to confirm and expand current findings.

## Data Availability

Not applicable.
